# Preliminary non‐comparative evaluation of an adapted colonoscopy preparation protocol for dogs using sodium picosulphate and magnesium citrate

**DOI:** 10.1002/vetr.5432

**Published:** 2025-08-26

**Authors:** Julia Elia da Silva Paranhos, Melissa Guillen Gonçalves de Souza, Daniela Araujo de Sousa, Debora Costabile Soibelman, Sabrina Martins da Costa Ferreira, Juliana da Silva Leite, Bruno Penna, Ana Maria Reis Ferreira

**Affiliations:** ^1^ Postgraduate Program in Veterinary Medicine (Clinical and Animal Reproduction) Universidade Federal Fluminense Niterói Rio de Janeiro Brazil; ^2^ Policlínica Veterinária Botafogo, Rua Oliveira Fausto Rio de Janeiro Rio de Janeiro Brazil; ^3^ Hospital Veterinário de Botafogo, Rua Diniz Cordeiro Rio de Janeiro Rio de Janeiro Brazil; ^4^ Intergavea, Rua Jardim Botânico, Jardim Botânico Rio de Janeiro Rio de Janeiro Brazil; ^5^ Department of Veterinary Clinic and Pathology, Laboratório de Anatomia Patológica Veterinária, Faculdade de Veterinária Universidade Federal Fluminense Niterói Rio de Janeiro Brazil; ^6^ Department of Microbiology and Parasitology, Laboratório de Cocos Gram Positivos, Instituto Biomédico Universidade Federal Fluminense Niterói Rio de Janeiro Brazil

**Keywords:** canine, endoscopy, enema, intestinal disease, laxative

## Abstract

**Background:**

Challenges associated with bowel cleansing protocols for canine colonoscopy include practical applicability and inadequate preparations. This study aimed to describe and evaluate an adapted protocol to simplify colonoscopy preparation in dogs.

**Methods:**

A low‐residue diet and a reduced volume of sodium picosulphate and magnesium citrate laxative were used orally alongside a saline enema. Forty‐eight dogs were included. Dogs received different volumes of the laxative and enema, according to their weight categories (<7 kg, 7‒20 kg and >20 kg). Bowel preparation was evaluated using the following scoring system: 0 for inadequate preparation, 1 for moderate preparation, 2 for good preparation and 3 for excellent preparation. Fisher's exact test was applied to assess the relationship between weight categories and preparation scores.

**Results:**

Two dogs (4%) had a preparation score of 1, 11 dogs (23%) had a score of 2 and 35 dogs (73%) had a score of 3. No dog had a score of 0. Two dogs (4%) experienced nausea. No statistically significant association was found between weight categories and preparation scores (*p* = 0.46).

**Limitations:**

The limitations of the study include the lack of a defined bowel preparation scale, no comparison with alternative protocols, no statistical assessment of score validation, the potential effects of anaesthetic drugs on bowel motility and the inability to assess interobserver variability when assigning preparation scores due to the use of a consensus approach.

**Conclusions:**

The present protocol for bowel cleansing in dogs before colonoscopy was both effective and easy to implement, with a low occurrence of adverse reactions.

## INTRODUCTION

Colonoscopy plays an important role in the diagnosis of colonic and rectal disease in dogs.^1^, ^2^ Lower bowel preparation for colonoscopy is essential to achieve clean mucosal surfaces, ensuring better diagnostic quality of the examination,^3^ but preparations are often disliked or poorly tolerated by human patients,^4^ and the same has been observed in veterinary patients.^5^ Factors such as the taste and volume of the laxative, along with unclear administration instructions, can affect the proper intake of the correct dose and result in inadequate bowel preparation,^4^ which may extend procedure times or lead to unsuccessful ileal visualisation and missed pathology.^6^

Solutions used for bowel preparations in people include orally administered agents such as polyethylene glycol electrolyte lavage solution (PEG‐ELS), polyethylene glycol (PEG) 3350, sodium sulphate and sodium picosulphate.^4^ Additionally, the use of a sodium phosphate‐based enema (e.g., Fleet's enema) has been widely documented for bowel preparation in people^7^, ^8^; however, caution is warranted when using these products in veterinary practice as they have been associated with toxicity in small animals.^9^ Although the administration of PEG usually requires the use of orogastric tubes and may cause adverse effects such as vomiting and fatal aspiration pneumonia, it is the most commonly described product for colonoscopy preparation in dogs.^3^, ^5^, ^10^, ^11^, ^12^, ^13^, ^14^, ^15^

The use of bowel‐cleansing products before colonoscopy in dogs was first described in 1989 using the laxative PEG,^10^, ^11^ an osmotic agent.^12^ Since these initial studies, many protocols have been described using other components, such as sodium phosphate‐based products, given orally or rectally as osmotic laxatives; bisacodyl, which acts as a stimulant laxative^3^, ^16^, ^17^; sodium picosulphate in combination with magnesium citrate, acting as both a stimulant and osmotic laxative^18^; and senna,^13^ a stimulant laxative derived from plants with laxative properties due to the action of sennosides and their active metabolite, rheinanthrone, in the colon.^19^ The use of warm‐water enemas, either alone or in combination with other bowel‐cleansing products, has also been widely described, with variable results achieved.^5^, ^11^, ^12^, ^13^, ^14^, ^15^ Table 1 outlines various components employed in canine bowel preparations and their potential adverse effects.

**TABLE 1:** Commonly described bowel preparations in dogs and their disadvantages and potential adverse effects

Product	Category	Route of administration	Authors	Disadvantages	Possible adverse effects
Bisacodyl	Stimulant laxative	Oral	Daugherty et al., 2008^3^ Trindade et al., 2009^16^	NDP	Vomiting and regurgitation, mild haematochezia^3^
PEG	Osmotic laxative	Oral	Burrows, 1989^10^ Richter and Cleveland, 1989^11^ Leib et al., 2004^5^ German et al., 2008^12^ Chamness, 2013^14^ Adamovich‐Rippe et al., 2017^15^ Zakerian et al., 2018^13^	Requires orogastric or nasogastric intubation and a large volume for administration.	Fatal aspiration pneumonia^5^ Vomiting after oral administration^13^ Self‐limiting dyspnoea and diarrhoea^15^
Senna	Stimulant laxative	Oral	Zakerian et al., 2018^13^	NDP	NDP
Sodium phosphate‐based products	Osmotic laxative	Oral	Daugherty et al., 2008^3^ Trindade et al., 2009^16^ Steffey et al., 2016^20^ Adamovich‐Rippe et al., 2017^15^	Inadequate bowel preparation compared to PEG and rectally administered phosphate‐based products.	Vomiting and regurgitation, mild haematochezia^3^ Increased respiratory rate, fever, arrhythmia^15^ Acute hypernatraemia and hypocalcaemia after oral administration^a^ ^,^ ^21^
		Rectal	Trindade et al., 2009^16^ Kalaiyarasan et al., 2023^17^	Tube for rectal administration may cause lesions in the colonic mucosa.	
Sodium picosulphate and magnesium citrate	Stimulant and osmotic laxative	Oral	de Souza and Botelho, 2019^18^	Large volume for oral administration (150 mL), especially in small dogs.	NDP
Warm water	Mechanical lavage	Rectal	Richter and Cleveland, 1989^11^ Leib et al., 2004^5^ Daugherty et al., 2008^3^ German et al., 2008^12^ Chamness, 2013^14^ Adamovich‐Rippe et al., 2017^15^ Zakerian et al., 2018^13^	Difficult for dog owners to perform at home. Tube for rectal administration may cause lesions in the colonic mucosa.	NDP

Abbreviations: NDP, no description provided; PEG, polyethylene glycol.

^a^ This publication specifically addresses an adverse reaction to the product and does not describe a bowel preparation protocol per se.

Sodium picosulphate acts as a laxative by stimulating the colon mucosa, while magnesium citrate works by retaining fluid in the colon, resulting in a combined stimulant and osmotic laxative effect for bowel cleansing. Sodium picosulphate is a prodrug that, upon hydrolysis by bacteria in the colon, is converted into its active metabolite, bis‐(p‐hydroxyphenyl)‐pyridyl‐2‐methane, which exerts its laxative effect by stimulating the transfer of fluids and electrolytes into the bowel lumen. It is commonly marketed in combination with magnesium oxide and citric acid to improve both its taste and cathartic action.^22^

Although the use of sodium picosulphate and magnesium citrate in combination with a low‐residue diet has demonstrated satisfactory results, as the laxative was reported to be easy to administer and effectively prepared the bowel for colonoscopy, resulting in a clear mucosal surface,^18^ a large volume of the dissolved laxative is still required for administration in dogs. Therefore, the aim of this study was to describe and evaluate a protocol that was adapted to simplify colonoscopy preparation in dogs.

## MATERIALS AND METHODS

### Inclusion and exclusion criteria

Dogs were included in the study regardless of their age, sex or breed, as long as they had a valid indication for undergoing colonoscopy. However, dogs presenting with renal impairment, such as azotaemia or proteinuria (evidenced by a high protein‐to‐creatinine urine ratio), as well as those with dehydration or electrolyte imbalances identified during preoperative screening, were excluded.

### Animals

A total of 48 dogs were included. All dogs included in the study were adults with no known pre‐existing conditions, apart from intestinal disease. All procedures occurred between 2020 and 2024 at the Universidade Federal Fluminense Veterinary Hospital (HUVET) and at two veterinary clinics in Rio de Janeiro, with the same preparation protocol used in each case. The primary indications for colonoscopy were inflammation and suspected colonic masses, which were based on gastrointestinal signs such as bleeding, diarrhoea, dyschezia or tenesmus that required investigation, and oncological follow‐up to monitor previously diagnosed neoplastic conditions.

### Colonoscopy preparation

In preparation for colonoscopy, dietary adjustments were necessary 2 days prior to the procedure. This involved the dog consuming only smooth‐textured food (commercial or homemade) without solid components 48 hours before the procedure and exclusively broth (liquid) on the day before. The broth was prepared by combining a single protein source (either chicken, fish or pork) with vegetables, simmering the mixture for 30 minutes, and subsequently straining out all solid components. Fasting from broth was required for 8 hours and from water for 6 hours before the procedure.

The laxative, Picoprep (Ferring Pharmaceuticals), containing the active ingredients sodium picosulphate (10 mg), magnesium oxide (3.5 g) and citric acid (12 g), along with excipients, was administered 12 and 4 hours before the procedure. The dosage instructions were as follows: for animals weighing under 7 kg, 8 g was mixed with 20 mL of water and administered orally at a dose of 1 mL/kg. For animals over 7 kg, 16.1 g was mixed with 20 mL of water and administered at the same dose. For animals over 20 kg, a maximum volume of 20 mL was recommended (see Table 2).

**TABLE 2:** Dose of laxative and enema volume used in the procedures, according to the dog's weight

Dog weight (kg)	Picoprep dissolved in 20 mL of water (g)	Oral dose	Enema (NaCl 0.9%) (mL)
<7	8	1 mL/kg	250
7‒20	16.1	1 mL/kg	500
>20	16.1	20 mL (max.)	500

Prior to the colonoscopy procedure but after anaesthesia, an enema of 250 mL of warm saline (NaCl 0.9%) for animals under 7 kg and 500 mL for animals over 7 kg was administered (see Table 2). A silicone catheter was connected to the saline infusion set and gently inserted into the colon to its maximum extent, allowing the saline to flow by gravity. The enema was administered as a continuous single flush until the liquid expelled from the rectum was clear.

### Colonoscopy procedure

Colonoscopy procedures were performed on 15 dogs at the Universidade Federal Fluminense Veterinary Hospital (HUVET), while 33 procedures were conducted at two external veterinary clinics. The procedures were carried out by three veterinarians, each with specialised training in small animal endoscopy, and the equipment used included the veterinary scopes Ultramedic V 1500 and Argus EV‐210 of 8.5 mm diameter and a human paediatric Fujinon 2500 scope of the same diameter.

The dogs received intramuscular acepromazine (0.02 mg/kg) and meperidine (2 mg/kg) as preanaesthetic medication, followed by a 5‐minute preoxygenation period with 100% oxygen before anaesthesia induction with 2‒5 mg/kg of propofol intravenously. Once clinical signs of unconsciousness were observed, endotracheal intubation was performed and the inhalational anaesthetic agent, isoflurane (1.2‒1.6%), was administered diluted in 100% oxygen. Should any adjustments to the anaesthetic protocol have been necessary, they were determined by a veterinary anaesthesiologist.

The dogs were placed in left lateral recumbency for colonoscopy, and all endoscopic procedures were performed according to the World Small Animal Veterinary Association standards.^23^ The ileocolic valve, ascending, transverse and descending colon, and rectum were evaluated in all animals.

### Scoring of bowel preparation

Images of the procedures were assessed at two different stages. Initially, each of the three endoscopists (D.C., J.E. and M.G.) was assigned to evaluate a specific subset of dogs during the colonoscopy procedure. Each endoscopist assigned a preparation score to the dogs they evaluated, and these scores were subsequently recorded in the endoscopy report. Following this, a review of the procedure images was conducted, where the images were independently assessed by the three veterinary endoscopists involved in the study (D.C., J.E. and M.G.), along with a fourth endoscopist (D.S.) from the group, who also had specialised training in small animal endoscopy and had over 10 years of experience.

**FIGURE 1:**
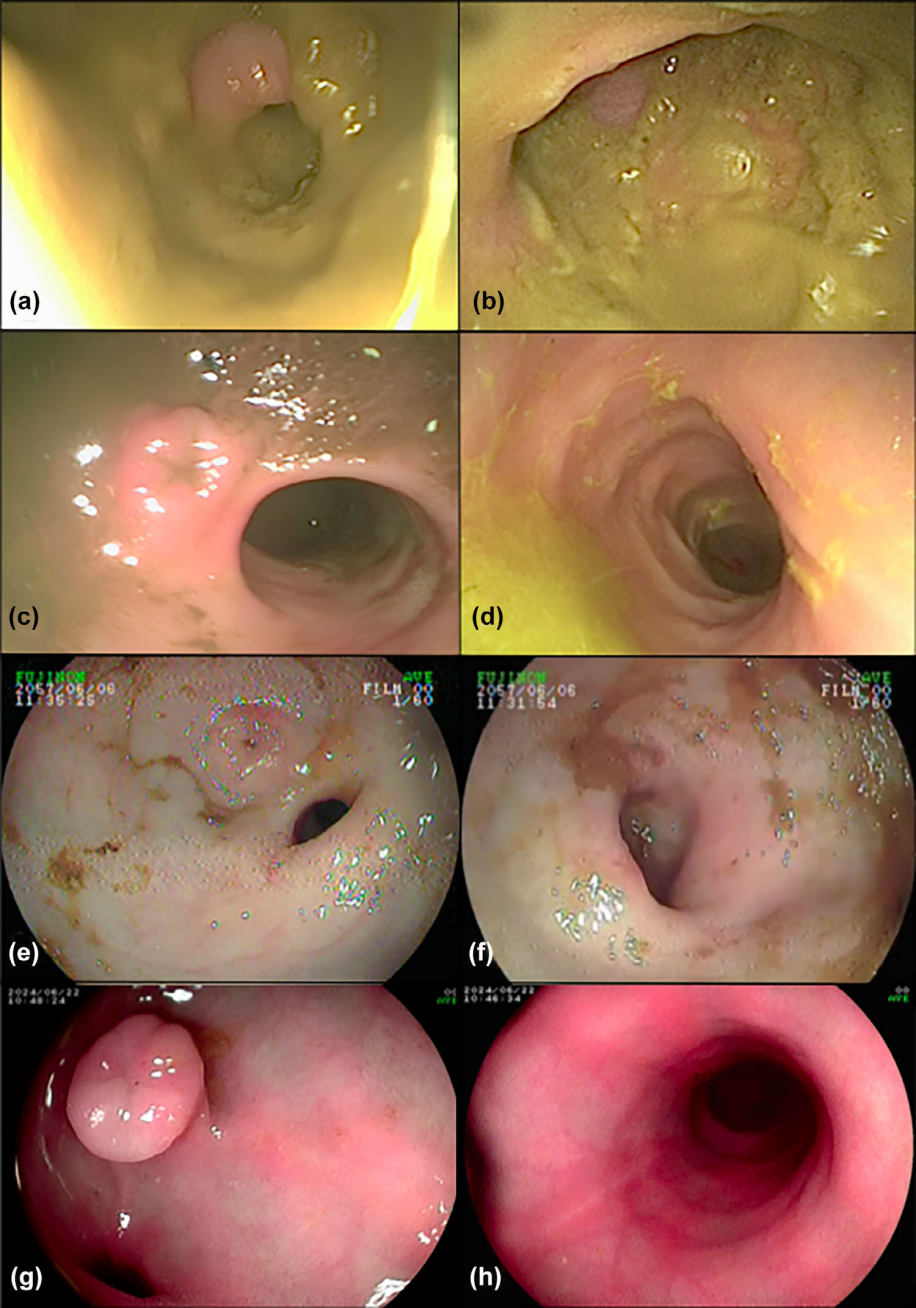
Endoscopic appearance of the ileocolic valve and colon mucosa in dogs. (a and b) Score of 0 from a dog not included in this study, used for comparison purposes; (c and d) score of 1; (e and f) score of 2; and (g and h) score of 3. All images, except for (a) and (b), are from dogs that participated in the study

The aim of this evaluation was to reach a consensus on the preparation score for each colonoscopy, and the final reported score for the study was the result of this consensus process. The evaluation of preparation involved assessing the presence of ileal effluent and faecal material within the lumen or adherent to the colonic mucosa, and was based partly on the human Boston Bowel Preparation Scale (BBPS),^24^ with scores assigned as follows: score 0 indicated inadequate preparation, characterised by a large amount of faecal material within the lumen or adherent to the colonic mucosa, preventing visualisation of the ileocolic valve; score 1 denoted moderate preparation, with a moderate amount of faecal material within the lumen or adherent to the colonic mucosa, with moderate ileal effluent; score 2 represented good preparation, with a small amount of faecal material within the lumen or adherent to the colonic mucosa, with small amounts of ileal effluent; and score 3 indicated excellent preparation, characterised by a clean mucosal surface with no ileal effluent present (Figure 1).

Scoring was always conducted after the colon had been washed with a saline enema and any excess liquid had been suctioned through the scope suctioning channel. The colon mucosa was initially evaluated in the descending colon, proceeding through the splenic flexure to the transverse colon, and then through the hepatic flexure to the ascending colon to the limit of the ileocolic valve. A single score was assigned based on the overall appearance of the mucosa across all regions, with the final score reflecting the worst score among the areas evaluated.

### Statistical analysis

Scores obtained with the preparation were evaluated using descriptive analysis. Fisher's exact test was applied to assess the relationship between weight categories (<7 kg, 7‒20 kg or >20 kg) and preparation scores, with the significance level set at 5%.

## RESULTS

Twenty of the 48 dogs were male (42%) and 28 were female (58%). Indications for colonoscopy included suspected chronic enteropathy in 38 dogs (79%), suspected neoplasia in eight dogs (17%) and oncological follow‐up to monitor previously diagnosed neoplastic conditions in two dogs (4%).

Of the 48 dogs, 18 (37.5%) weighed under 7 kg, 22 (45.8%) weighed between 7 and 20 kg, and eight (16.7%) weighed more than 20 kg. Two dogs (4%) had a preparation score of 1, 11 dogs (23%) had a preparation score of 2 and 35 dogs (73%) had a preparation score of 3 (Figure 1). No dog had an inadequate preparation score (score 0). No statistically significant association was found between weight categories and preparation scores (*p* = 0.46).

Two dogs (4%) experienced nausea following the preparation but were able to complete it, with no vomiting or other clinical signs observed. One dog vomited immediately after administration and, as it was unable to complete the preparation, the colonoscopy was not performed, and the dog was excluded from the study.

## DISCUSSION

Widely recognised, validated and frequently used bowel preparation scoring systems in human medicine are the Aronchick Scale,^25^ the Ottawa Bowel Preparation Scale^26^ and the BBPS.^24^ In veterinary medicine, there is currently no universally accepted scoring system for bowel preparation. Nevertheless, Daugherty et al.^3^ introduced a 1‒4 scoring system, Trindade et al.^16^ and de Souza and Botelho^18^ used a grade I‒IV system to evaluate colonoscopy preparation in dogs and Zakerian et al.^13^ used a 1‒4 scoring system to assess colonic cleansing for colonoscopy. Many of these veterinary scoring systems are adaptations of human systems.

In the study by Daugherty et al.,^3^ five regions of the colon (the distal, mid‐portion and orad portion of the descending colon, the transverse colon and the ascending colon) were scored, and the scoring system included two descriptions of ‘clean colon’: grade 1 for a clean colon with no faecal matter (or nearly none) and no residual fluid, and grade 2 for a clean colon with small amounts of thin, adherent liquid faecal matter that could be easily suctioned or flushed. The system also included two descriptions of ‘unacceptable preparation’: grade 3 for moderate amounts of liquid to semi‐solid or adherent faecal matter that were difficult to suction or flush, with the mucosa still visible, and grade 4 for large amounts of solid or adherent faecal matter that precluded adequate examination. Zakerian et al.^13^ adopted a classification system similar to that of Daugherty et al.,^3^ separately evaluating five regions of the colon—distal, mid and orad portions of the descending colon, transverse colon and ascending colon—and calculating a total score based on the sum of the regional scores, with scores ranging from 1 (clean colon) to 4 (unacceptable preparation). Trindade et al.^16^ and de Souza and Botelho^18^ also employed a four‐grade system, classifying preparations from grade I (excellent, with no faeces) to grade IV (poor preparation).

In our investigation, the grading system involved assessing the appearance of the colon mucosa and ileal effluent subsequent to colon cleansing via enema administration. Scores ranged from 0, indicative of inadequate preparation, to 3, indicative of excellent preparation, similar to the human BBPS. None of the dogs included in our study were assigned a preparation score of 0. However, the score of 0 was defined based on the appearance of the colon, as observed by our group in previous cases of unsuccessful bowel preparations (Figure 1a,b). Moreover, in line with the BBPS human protocol, the assessment was conducted after colon washing and suctioning, as this enhances mucosal visibility for scoring by removing fluid and semi‐solid debris.^24^ Although the BBPS system divides the colon into distinct segments for scoring, our visual observations of the colon appearance showed no noticeable differences between the regions. These observations were not statistically assessed, as formal comparisons between the regions were not performed; hence, we assigned scores based on the overall condition of the colon mucosa.

The administration of sodium picosulphate, alongside fasting and dietary modifications, is recommended to optimise colonoscopy preparation in dogs.^18^ In the study by de Souza and Botelho,^18^ the effectiveness of Picoprep was evaluated in 10 adult dogs. The dogs were given an oral dose of 150 mL of diluted Picoprep 24 and 12 hours prior to the colonoscopy procedure, which resulted in a clear mucosal surface without any reported adverse effects. In people, the timing of Picoprep administration varies depending on the scheduled appointment time. According to the manufacturer's instructions, for morning colonoscopies, the first dose should be taken in the late afternoon of the day before, followed by the second dose 6 hours later. For afternoon procedures, the first dose is taken the night before, with the second dose taken on the morning of the procedure. In our study, we adapted the administration schedule by giving dogs the first dose of Picoprep 12 hours before the colonoscopy procedure, followed by a second dose 4 hours before the examination. This adjustment was deemed more convenient for owners to adhere to and showed favourable outcomes.

To mitigate potential adverse reactions, de Souza and Botelho^18^ recommended the administration of oral scopolamine, dipyrone and metoclopramide 3 days prior to the colonoscopy, likely contributing to the absence of observed adverse reactions. However, in our study, no medication other than the laxative was prescribed. Two dogs experienced nausea, but they were able to complete the preparation without any further issues, and no other clinical signs or abnormal behaviour were noted; however, one dog vomited immediately after receiving Picoprep. This dog was subsequently excluded from the study and was further diagnosed with large granular lymphocyte lymphoma. Therefore, it is difficult to ascertain whether the vomiting was attributable to the dog's underlying condition, given its history of recurrent nausea and emesis associated with lymphoma, or the use of the laxative. According to the Picoprep summary of product characteristics, human patients may commonly experience symptoms such as nausea, abdominal distension and bloating. Less frequently, patients may also experience abdominal cramps, vomiting and anal irritation.

Furthermore, the endeavour to use a more concentrated solution, achieved by diluting the laxative in a reduced volume of water (20 mL), resulted in a markedly smaller volume for oral administration, making it easier for the dogs to consume the laxative. A high level of compliance with the protocol was noted, since when asked about the preparation process, all owners confirmed that their dogs had taken the laxative without any issues and found it easy to administer. Previous research investigating the oral administration of sodium phosphate solutions in dogs has highlighted the benefits of using lower volumes of laxative compared to PEG solutions.^3^, ^16^ Nonetheless, despite these advantages, PEG preparations have been deemed superior to sodium phosphate solutions.^3^ Another benefit noted in some dogs with our modified protocol was the occurrence of diarrhoea primarily on the morning of the procedure, often while the dog was already at the clinic, thus alleviating concerns typically reported by owners about the laxative triggering diarrhoea at home.

It is important to highlight that Picoprep is contraindicated in human patients with renal impairment due to the potential risk of magnesium accumulation in the plasma, as well as in cases of gastrointestinal obstruction, gastric retention, bowel perforation, toxic megacolon or colitis, ileus, patients with a stoma or those with hypersensitivity to its ingredients. Consequently, cautious administration of Picoprep is warranted in dogs, particularly given that the only previously published study in veterinary medicine has primarily used the laxative in adult dogs without any comorbidities.^18^

Various fasting intervals have been documented for canine colonoscopy in the literature, ranging from 12 hours^18^ to 24,^16^, ^17^, ^27^ 36^20^ and 72 hours.^1^, ^2^ In our investigation, we proposed cessation of broth consumption 8 hours before the procedure, with water withdrawal initiated 6 hours before colonoscopy. Our observation revealed a high rate of acceptance of these instructions by dog owners and their animals, which helped avoid prolonged periods of unnecessary fasting and supported hydration and electrolyte intake. Additionally, we permitted the provision of natural coconut water; however, recognising its limited availability globally, offering commercial pet electrolyte solutions is a viable alternative.

Currently, there is no consensus on the use of enemas for bowel cleansing. Warm water enemas are well documented,^3^, ^5^, ^11^, ^12^, ^13^, ^14^, ^15^ whereas saline enemas have less documentation.^28^, ^29^ Concerns about potential irritation of the colon from other ingredients have led to a preference for warm water^2^; however, we found no contraindications or adverse effects associated with the use of warm saline enemas. A large volume of saline was used in our study to thoroughly flush the colon and this approach is consistent with previous descriptions of enema volumes used in colonoscopy preparation, which employed doses of 50 mL/kg or greater.^30^ Concerns about the potential risks of high‐volume saline administration rectally were mitigated, as much of the saline was expelled rather than absorbed by the dog during the procedure. No complications were observed during the administration of the enema or following the colonoscopy procedure.

Regarding the anaesthetic protocol, most dogs received the same regimen; however, in some cases, an individualised approach was necessary, requiring the use of alternative drugs. Anaesthetic agents can influence intestinal motility and sphincter function, which may limit the effectiveness of certain procedures,^31^ such as the administration of an enema. Nevertheless, despite the potential effects of anaesthesia, our preparation protocol appeared effective, and the decision to perform the enema under anaesthesia was made for practical reasons.

While this study offers valuable insights, a few limitations should be noted. Ileoscopy was not possible in all dogs due to challenges with passing the ileocolic valve, considering variations in dog size and the diameter of the endoscope used. As a result, we focused on colon evaluation to ensure standardisation of the findings. Additionally, the scoring system used was not statistically assessed, and colonoscopy videos were not available for re‐evaluation in some cases; therefore, only images were used. The use of a consensus scoring approach, while beneficial for standardising assessments, may have limited the potential for detecting variability in individual observer assessments. These factors should be considered when interpreting the findings, and future research could further explore these areas to strengthen the application of the scoring system.

## CONCLUSION

In conclusion, our adapted protocol, which combines a low‐residue diet with the oral administration of a reduced volume of sodium picosulphate and magnesium citrate laxative solution and regular saline enema, proved to be both effective and easy to implement for bowel cleansing in dogs before colonoscopy, with a low occurrence of adverse reactions.

## AUTHOR CONTRIBUTIONS

*Conceptualisation*: Julia Elia da Silva Paranhos and Melissa Guillen Gonçalves de Souza. *Methodology*: Julia Elia da Silva Paranhos; Melissa Guillen Gonçalves de Souza; Debora Costabile Soibelman; Daniela Araujo de Sousa and Sabrina Martins da Costa Ferreira. *Formal analysis*: Julia Elia da Silva Paranhos; Daniela Araujo de Sousa; Juliana da Silva Leite; Bruno de Araujo Penna and Ana Maria Reis Ferreira. *Investigation*: Julia Elia da Silva Paranhos and Daniela Araujo de Sousa. *Writing*: Julia Elia da Silva Paranhos; Melissa Guillen Gonçalves de Souza and Daniela Araujo de Sousa. *Supervision*: Juliana da Silva Leite; Bruno de Araujo Penna and Ana Maria Reis Ferreira. All the authors have read and agreed to the published version of the manuscript.

## CONFLICT OF INTEREST STATEMENT

The authors declare they have no conflicts of interest.

## ETHICS STATEMENT

This study was approved by the Ethics Committee on Animal Use (Comitê de Ética no Uso de Animais of Universidade Federal Fluminense) under the protocol number 3847070623. All applicable international, national and/or institutional guidelines for the care and use of animals were followed. All procedures were performed with the owner's consent.

## Data Availability

The data that support the findings of this study are available from the corresponding author upon reasonable request.
